# Power consumption for an agitated vessel equipped with pitched blade turbine and short baffles

**DOI:** 10.1007/s11696-017-0346-x

**Published:** 2017-11-28

**Authors:** Marta Major-Godlewska, Joanna Karcz

**Affiliations:** Department of Chemical Engineering, West Pomeranian University of Technology, al. Piastów 42, 71-065 Szczecin, Poland

**Keywords:** Power consumption, Agitated vessel, Pitched blade turbine, Short baffle

## Abstract

Power characteristics for an agitated vessel equipped with planar short baffles of length *L* and pitched blade turbine of pitch *β* are presented in the paper. The studies were carried out in the vessel of inner diameter *D* = 0.6 m, where the baffles were located in the distance *p* from the vessel bottom (*p* + *L* = *H*). Torque was measured using strain gauge method within the turbulent regime of the flow of Newtonian liquid in the agitated vessel. The effects of the pitch *β* and geometrical parameter *p*/*H* on the power number *Ne* were determined mathematically. The results showed that, for the assumed value of the angle *β*, the function *Ne* = *f* (*L*/*H*) decreases with the decrease in the baffle length *L* (i.e. with the increase in the parameter *p*). Moreover, for the assumed value of the baffle length *L*, the function *Ne* = *f* (*β*) increases with the increase in the angle *β* of the inclination of the impeller blade.

## Introduction

Liquids of low viscosity are usually agitated in the vessels of inner diameter *D*, equipped with the high-speed impeller and four (*J* = 4) planar baffles of width *B* = 0.1 *D* and length *L* = *H*. Baffling effect can be described by the dimensionless parameter *J* × (*B*/*D*) × (*L*/*H*), which is equal to 0.4 for the standard geometry of the baffled agitated vessel (i.e. *D* = *H*; *J* = 4; *B*/*D* = 0.1; *L*/*H* = 1). Power characteristics for the systems agitator—planar baffles of full length—vessel system within the wide range of the *Re* numbers are presented in the literature by many authors (Rushton et al. 1950; Nagata 1975); Stręk 1981; Oldshue 1983; Harnby et al. 1992).

Effects of the geometrical parameters of the baffles of full length on the agitation efficiency were tested experimentally and numerically. Lu et al. (1997) examined the effects of width *B* and number *J* of baffles in the agitated vessel with single or triple standard Rushton turbine for systems with and without aeration. The authors proved that insertion of the appropriate number of baffles improves the extent of liquid mixing. However, the excessive baffling and sparging gas through the impeller would interrupt the liquid mixing and increase the mixing time. Hashimoto et al. (2011) investigated the baffling effect on the enhancement of mixing under a laminar condition in the vessel with two-bladed paddle impeller and two baffles. Their results showed that baffles can effectively transform the circumferential flow to vertical and radial flows. Kamla et al. (2016) carried out CFD study on the effect of baffles of full length on the energy consumption and the flow structure in an agitated vessel equipped with the Rushton turbine. The authors tested the effect of the baffle curvature at the system with four baffles of width *B* = 0.1*D*. Each baffle was in shape of the annulus sector situated vertically in relation to the cylindrical vessel wall. Kamla et al. (2016) concluded that power consumption decreases with the increase in the radius of the baffle curvature for the clockwise rotational direction of the impeller. Heyter and Wollny (2017) examined the effect of different baffle variations on the agitation efficiency in the vessels equipped with axial multi-impeller system in order to determine power number. The experiments were carried out in the vessel of inner diameter *D* = 0.4 m filled by the liquid up to height *H* = (1–1.5)*D*. The system of pitched blade turbines (*Z* = 45; *β* = 45^o^) of diameter *d* = 0.605*D* was used. Planar baffles of full length were arranged under the angle equal to 90^o^ or 60 ^o^ in relation to the cylindrical wall of the vessel. The measurements were taken within the range of the baffle numbers 1 ≤ *J* ≤ 4 and baffle width *B* = 0.08*D* or 0.1*D*.

Shorter baffles of the length *L* < *H* may also be recommended for the same applications, for example, for the optimization of the heat transfer process (Karcz and Stręk 1995), as well as for the dispersion of gas bubbles (Hsu et al. 1998) or the suspension of the heavy (Heywood et al. 1991; Karcz and Więch 1999) or floating (Karcz and Mackiewicz 2009) particles into agitated liquid.

Location of the short baffles in an agitated vessel, i.e. the distance p between bottom of the vessel and lower edge of the baffle (where *H* = *L* + *p*), depends on the type of the process, type of the physical system agitated (liquid phase, gas–liquid, solid–liquid or gas–solid–liquid system) and type of the interaction between impeller used (axial flow or radial flow mode) and short baffles. In case of the heat transfer process in a jacketed baffled agitated vessel, the most effective results (Karcz and Stręk 1995) are gained in the vessel with 8 short baffles (*L*/*H* = 0.25; *p*/*H* = 0.37) and equipped with the disc turbine of diameter *d* = 0.5*D* (*Z* = 10; *a*/*d* = 0.25; *b*/*d* = 0.2) located at height *h* = 0.5*H*. Hsu et al. (1998) experimentally tested power consumption for the gas–liquid system produced in the agitated vessel (*D* = 0.29 m; *H*/*D* ϵ < 1.34; 2 >) with two down—pumping pitched blade turbines (*Z* = 4; pitch *β* = 45^o^) and four shortened and narrower baffles (*L*/*D* = 1; *p* = 0; *B*/*D* ϵ < 1/30; 1/15 >) than standard ones. Hsu et al. (1998) achieved a steady gas induction at lower impeller speeds and smaller power consumption as compared with the results for the agitated vessel with baffles of full length.

Heywood et al. (1991) investigated the effect of the short baffles on the production of the heavy particles suspension in the agitated vessel. The authors varied the baffle off-bottom clearance *p* only. Heywood et al. (1991) concluded that at *p* = 0.55*H*, the Rushton turbine requires one-fifth of the power consumption to attain complete suspension, in comparison with the standard configuration of baffles. Karcz and Mackiewicz (2009) experimentally studied effects of vessel baffling on the drawdown of floating solids. The measurements were taken in the agitated vessel (*D* = *H* = 0.3 m) with the high-speed impeller located at the height *h* = 0.33*H* or *h* = 0.67*H* and short baffles. Upper edge of the short baffles always corresponded to the height *H*, whereas distance between vessel bottom and lower edge of the baffle was equal to *p* = *H*–*L*. The results of the study showed that baffling effect on the just draw down agitator speed *n*_JD_ depends on the position *h* of the agitator in the vessel. Moreover, baffling effect found for the up-pumping pitched blade turbine position equal to *h* = 0.67*H* is significantly lower than the one which was obtained for the standard height of this agitator in the vessel (*h* = 0.33*H*).

Studies of the power consumption in the agitated vessel equipped with the short baffles and propeller or different types of the turbines [Rushton turbine (*Z* = 6); Smith turbine (*Z* = 6); pitched blade turbine (*Z* = 3; *β* = 45^o^)] were carried out by (Stręk and Karcz 1993 and Karcz and Major 1998). Up till now, the effect of the pitch of turbine blade on the power number has not been experimentally analysed.

The effects of impeller blade inclination on the process characteristics for un-baffled and baffled agitated vessel were studied by Suzukawa et al. (2006), Scargiali et al. (2014) and Ameur (2016). Using laser Doppler velocimetry method (LDV), Suzukawa et al. (2006) studied the effect of the blade attack angle on the roll and trailing vortex structures in a full baffled agitated vessel (*D* = 0.49 m) equipped with four-bladed paddle impeller of blade inclination equal to 45^o^, 60^o^, 75^o^ or 90^o^. Scargiali et al. (2014) experimentally studied the influence of impeller geometry on mass transfer process and power consumption of an un-baffled agitated vessel (*D* = 0.19 m) operating both in sub-critical conditions (i.e. the free surface vortex has not yet reached the impeller) and in super-critical conditions (i.e. the free surface vortex has reached the impeller). The authors stated that, among the impeller geometries tested, pitched blade turbine provided the most interesting oxygen transfer performance in the sub-critical regime, and therefore, it can be recommended for the agitation of the shear sensitive cultures. Using method of numerical analysis, Houari Ameur (2016) studied the effect of blade attack angle on the process characteristics for the shear thinning fluid agitated in an un-baffled agitated vessel equipped with two-bladed impeller of blade inclination equal to 45^o^, 60^o^, 75^o^ or 90^o^. Ameur (2016) proved that the power number and the cavern size increased with the increase in both the blade attack angle and shear thinning behaviour of the fluid.

In the paper, the results of the systematic measurements on the power consumption for an agitated vessel with the short baffles and six blade pitched blade turbine (PBT) with various angle *β* of the blade inclination have been presented.

## Experimental

**Fig. 1:**
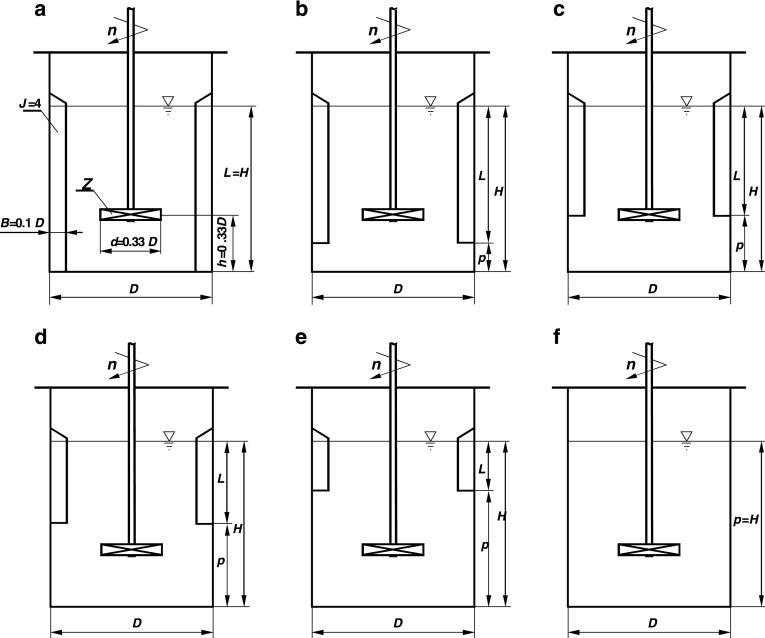
Arrangement of planar baffles in the agitated vessel: **a** *p/H* = 0; **b** *p/H* = 0.17; **c** *p/H* = 0.33; **d** *p/H* = 0.5; **e** *p/H* = 0.67; **f** *p/H* = 1

**Fig. 2:**
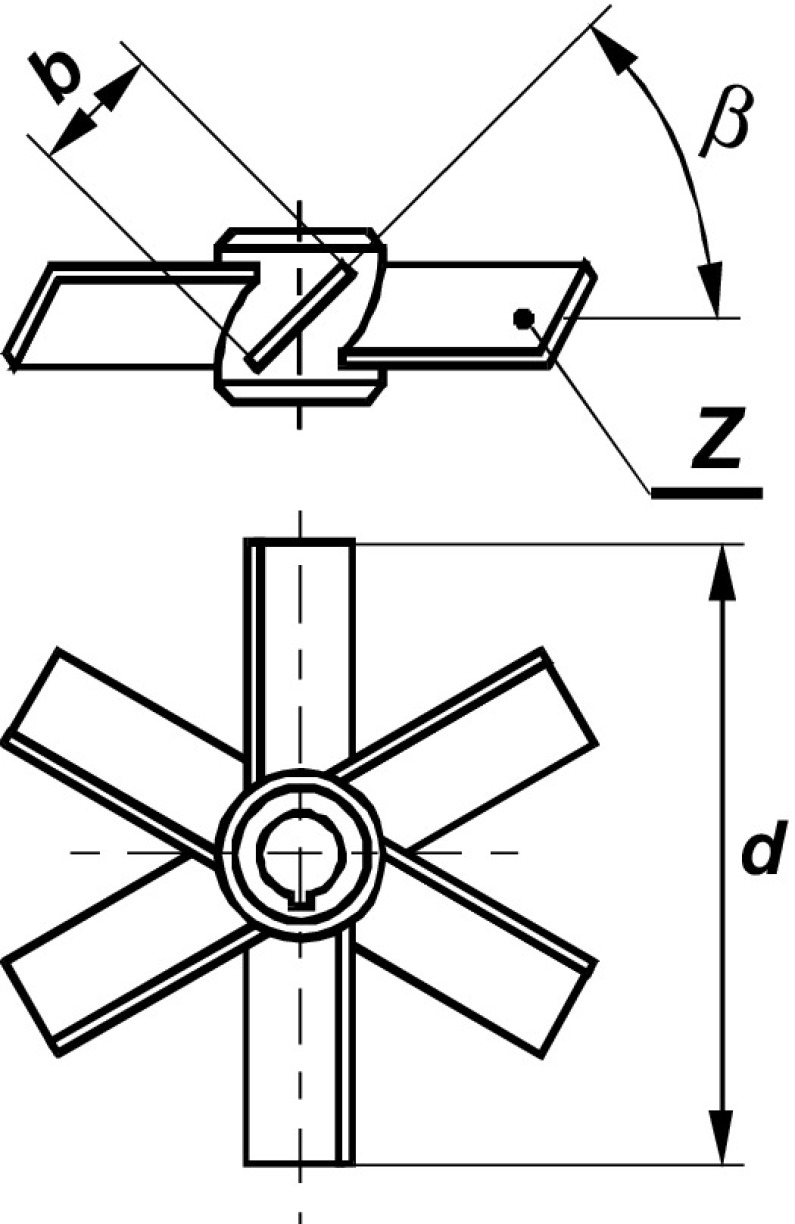
Pitched blade turbine used in the experiments: **a** *β* = 90^o^; *β* = 60^o^; *β* = 45^o^; *β* = 30^o^

The experiments of the power consumption were carried out in the agitated vessel of inner diameter *D* = 0.6 m, filled with the tap water up to height *H* = *D*. Four planar baffles of width *B* = 0.1*D* and length *L* were placed in the distance *p* from the flat bottom of the vessel. As Fig. 1 shows, the upper edge of the baffle was always located at the level of the free surface of the liquid and the geometrical constraint *p* + *L* = *H* was fulfilled. The geometrical parameters *p*/*H* and *L*/*H* were varied within the ranges *p*/*H* ∈ < 0; 1 > and *L*/*H*∈ < 1; 0 >, respectively, i.e. six series of the measurements for the *p*/*H* = 0; 0.17; 0.33; 0.5; 0.67 and 1 (*L*/*H* = 1; 0.83; 0.67; 0.5; 0.33; 0) were performed. The geometrical parameters *p*/*H* = 0 and *L*/*H* = 1 correspond to the geometry of the agitated vessel with standard baffles (Fig. 1a), whereas the parameters *p*/*H* = 1 and *L*/*H* = 0 describe the vessel without baffles (Fig. 1f). The pitched blade turbine of diameter *d* = 0.33*D* was mounted on the shaft with the clearance *h* = 0.33*D* from the bottom of the agitated vessel. The up-pumping pitched blade turbines with the six blades (*Z* = 6) and four different inclinations to the horizontal blade angle *β* = 90^o^, 60^o^, 45^o^ and 30^o^ (Fig. 2) were used in the tests. The measurements were taken within the turbulent regime of the liquid flow in the agitated vessel.

**Fig. 3:**
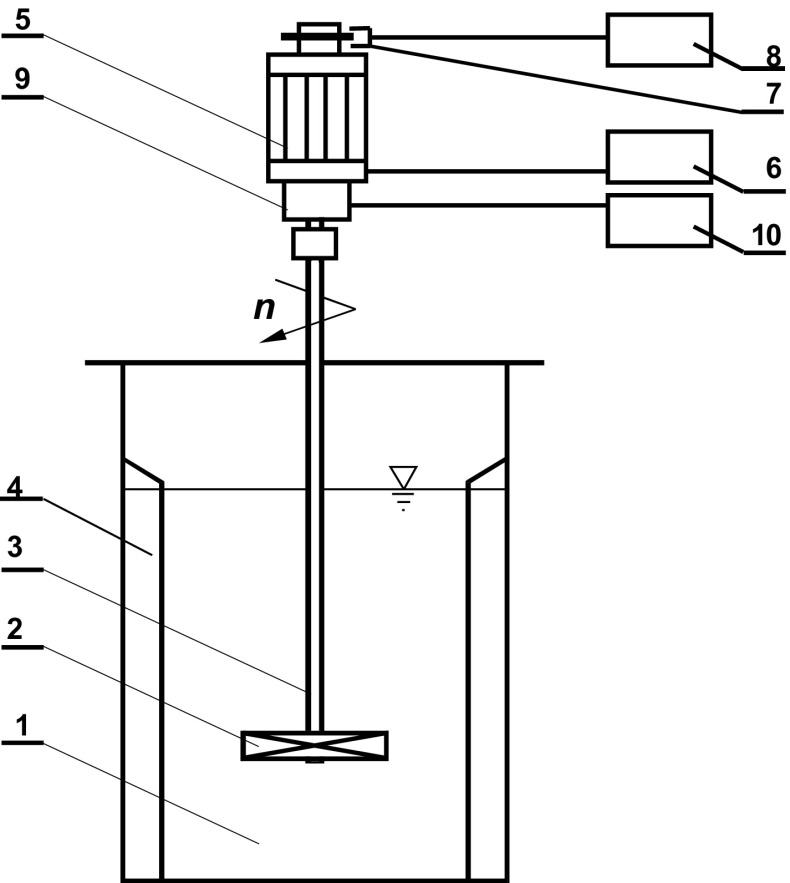
Experimental set-up: 1—agitated vessel; 2—agitator; 3—shaft; 4—baffle; 5—electromotor; 6—steering device; 7—photoelectric sensor; 8—electronic counter; 9—torsional sleeve with strain gauge and slip rings; 10—amplifier MGC

The strain gauge method was used for the measurement of the torque *M*. Power consumption *P* was calculated from the equation.

1 $$P = 2\pi n \, M,$$

where *n* [s^−1^]—impeller speed and *M* [Nm]—torque. Experimental set-up is shown in Fig. 3. The measurements were taken in the agitated vessel: (1) with the flat bottom, planar short baffles (4) and up-pumping pitched blade turbine (2). The turbine was driven by an electromotor (5) coupled with the steering device (6). The system for the measurement of the torque consisted of the torsional sleeve with the strain gauges and slip rings (9), which were connected with an amplifier MGC (10). The agitator speeds were determined by means of the photoelectric method with the photoelectric sensor (7) and an electronic counter (8) as the basic parts of the measuring system.

## Results and discussion

**Fig. 4:**
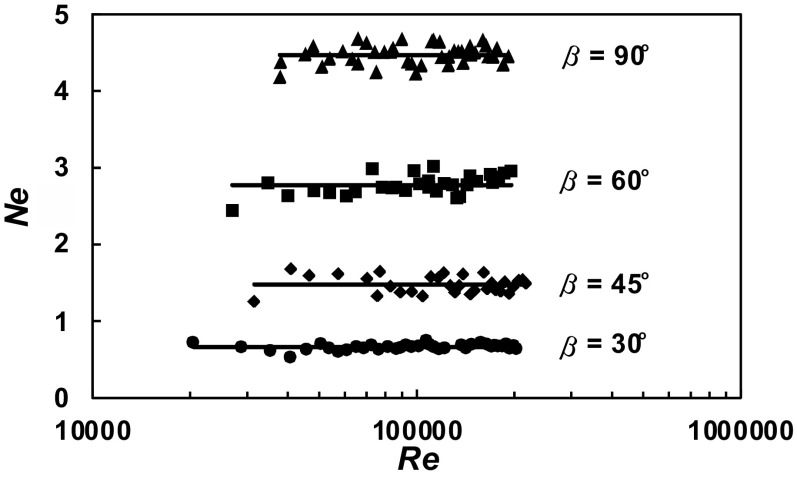
Power characteristics *Ne* = *f* (*Re*) for *p/H* = 0 and different blades turbines *β* = 90^o^; 60^o^; 45^o^; 30^o^

**Fig. 5:**
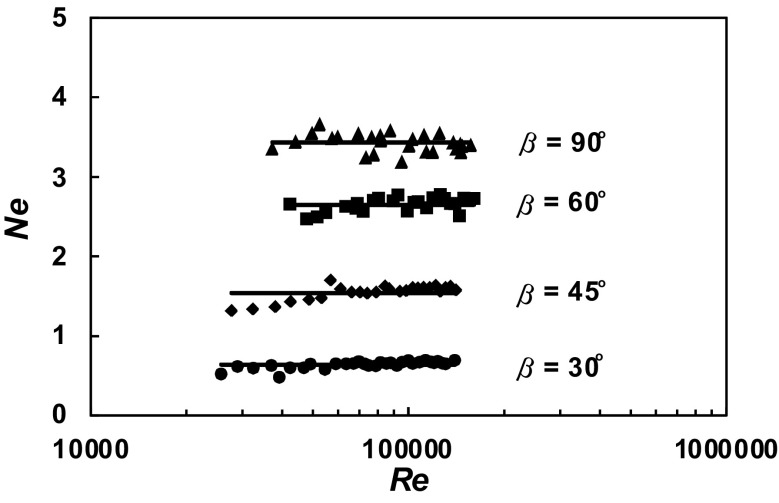
Power characteristics *Ne* = *f* (*Re*) for *p/H* = 0.17 and different blades turbines *β* = 90^o^; 60^o^; 45^o^; 30^o^

**Fig. 6:**
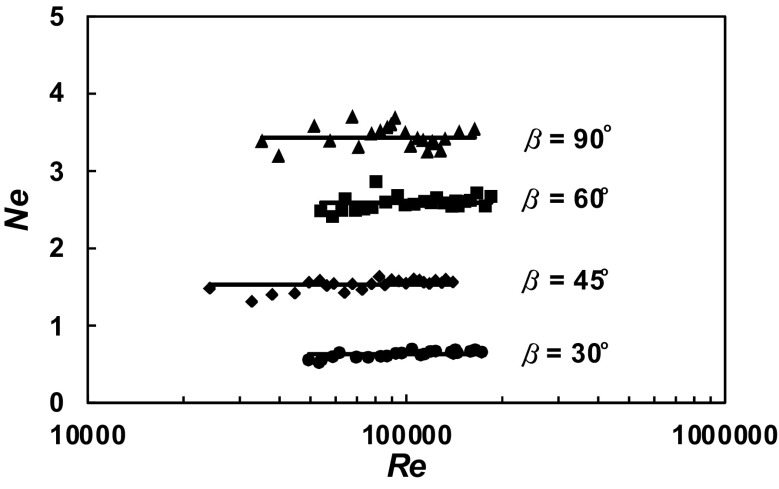
Power characteristics *Ne* = *f* (*Re*) for *p/H* = 0.33 and different blades turbines *β* = 90^o^; 60^o^; 45^o^; 30^o^

**Fig. 7:**
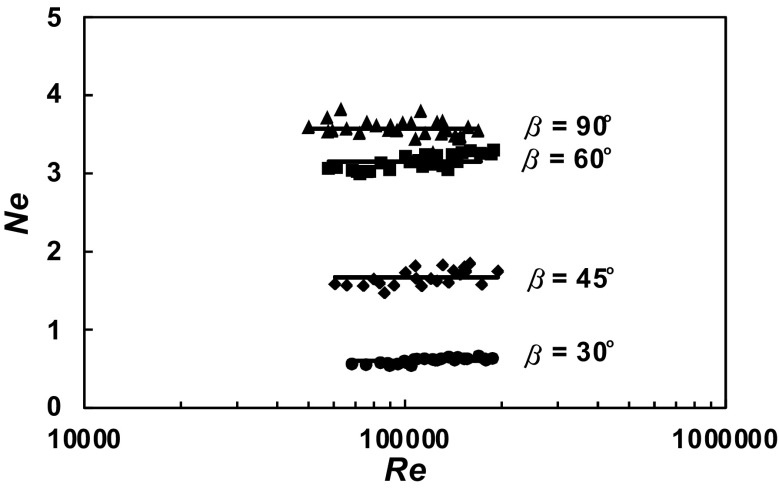
Power characteristics *Ne* = *f* (*Re*) for *p/H* = 0.5 and different blades turbines *β* = 90^o^; 60^o^; 45^o^; 30^o^

**Fig. 8:**
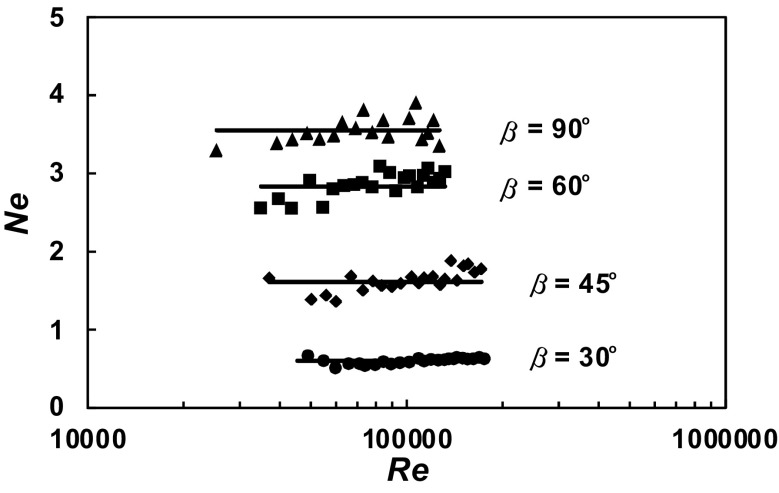
Power characteristics *Ne* = *f* (*Re*) for *p/H* = 0.67 and different blades turbines *β* = 90^o^; 60^o^; 45^o^; 30^o^

**Fig. 9:**
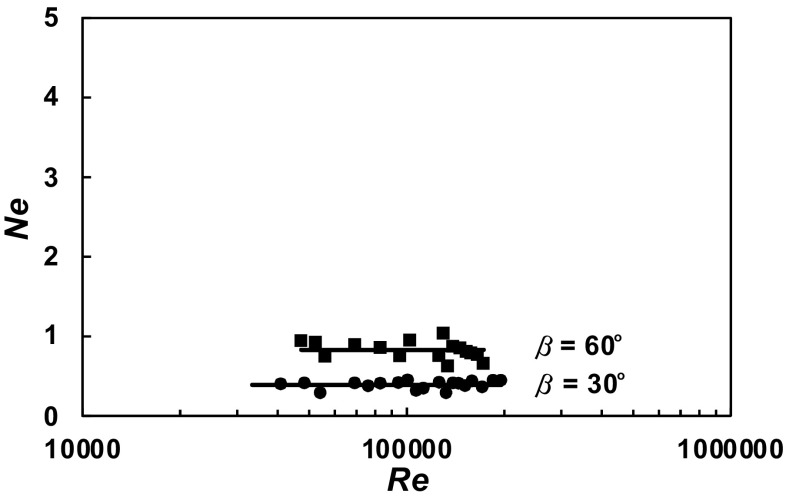
Power characteristics *Ne* = *f* (*Re*) for *p/H* = 1 and different blades turbines *β* = 60^o^; 30^o^

**Fig. 10:**
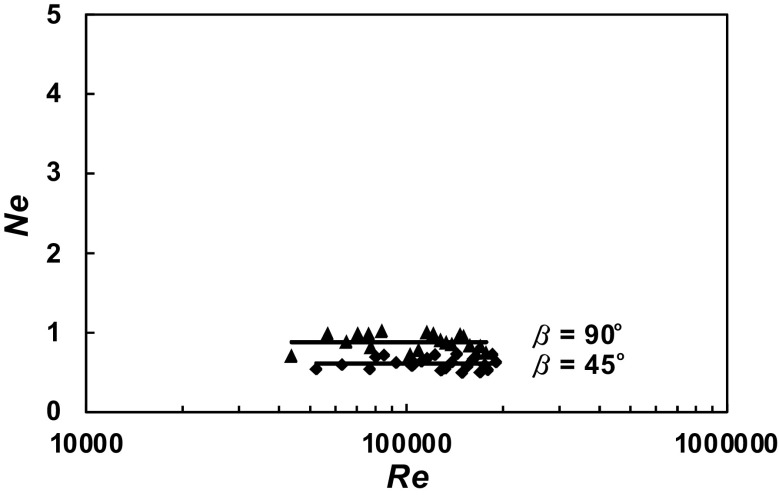
Power characteristics *Ne* = *f* (*Re*) for *p/H* = 1 and different blades turbines *β* = 90^o^; 45^o^

A total of 630 experimental data *P* = *f* (*n*) were obtained within the range of the *Re ∈* < 3 × 10^4^; 2 × 10^5^ > for the agitated vessel equipped with baffles of length *L* (*L*/*H ∈* < 1; 0 >; *p*/*H ∈* < 0; 1 >) and pitched blade turbine with the angle *β* ϵ < 90^o^; 30^o^ >. From these data, power characteristics

2 $$Ne = f\left( {Re} \right),$$

where

3 $$Ne = \frac{P}{{n^{3} d^{5} \rho }};Re = \frac{{nd^{2} \rho }}{\eta }$$

were determined for each geometry of the baffles-pitched blade turbine. The dependences *Ne* = *f* (*Re*) for a given parameter *p*/*H* = const and an angle *β* = const are presented in Figs. 4, 5, 6, 7, 8, 9, 10. The greatest power numbers *Ne* correspond to the turbine = 90^o^ and the values of the *Ne* decrease with the decrease in the angle *β* of the inclination of the impeller blade. The strongest effect of the angle *β* on the power number *Ne* was found for the agitated vessel equipped with the standard baffles [*L*/*H* = 1; *p*/*H* = 0 (Fig. 4)], where the values of *Ne* decrease five times with the change in the *β* within the limits (90^o^; 30^o^). As Figs. 9 and 10 show, the least of all, the power numbers *Ne* depend on the angle *β* for the agitated vessel without baffles. Mean values of the power numbers *Ne* for the agitated vessel equipped with four baffles of length *L* and pitched blade turbine of pitch *β* are given in Table 1.

**Table 1:** Mean values of power numbers *Ne* for agitated vessel equipped with pitched blade turbine of pitch and four baffles of length *L* (Re ϵ < 3 × 10^4^; 2 × 10^5^ >)

No.	*p/H*	*β*/deg	90	60	45	30
		*L/H*		*Ne*		
1	0	1	4.45	2.77	1.48	0.67
2	0.17	0.83	3.43	2.65	1.54	0.64
3	0.33	0.67	3.43	2.59	1.53	0.63
4	0.5	0.5	3.57	3.15	1.67	0.60
5	0.67	0.33	3.55	2.83	1.61	0.60
6	1	0	0.88	0.83	0.61	0.39

The results of the power consumption obtained in the non-baffled vessel (*p/H* = 1), shown in Fig. 10 and in Table 1 (No 6), can be compared to the data for PBT 45^o^ analysed in paper Scargiali et al. Scargiali et al. took the measurements in the agitated vessel of inner diameter *D* = 0.19 m, and they stated that critical rotational speed for PBT (assuming six blades of the impeller) at which the free surface vortex reaches the impeller is equal to 10.8 1/s. It corresponds to *Re* = 4.35 × 10^4^ and *Ne* = 0.52. In our case, up-pumping pitched blade turbine 45^o^ operated in the agitated vessel of inner diameter *D* = 0.6 m. For the highest Reynolds number *Re* = 2 × 10^5^, the agitator speed had the value *n* = 5 1/s what corresponded to power number *Ne* = 0.61. The differences between our *Ne* and *Re* numbers and those obtained by Scargiali et al. can be caused by different scale of both compared agitated vessels.

**Fig. 11:**
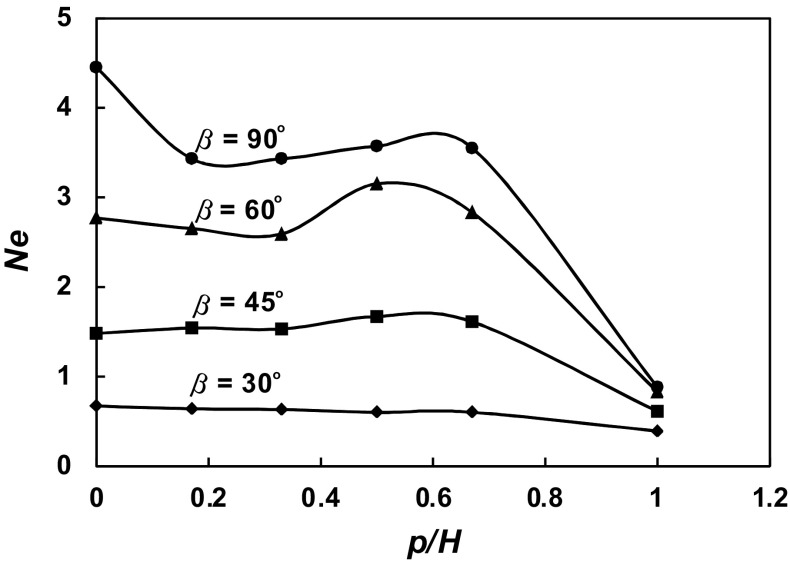
Dependence *Ne* = *f* (*p/H*) for different blades turbines *β* = 90^o^; 60^o^; 45^o^; 30^o^

The dependences of the power number *Ne* on the geometrical parameter *p*/*H* for different pitched blade turbines are presented graphically in Fig. 11. For the agitated vessel equipped with the pitched blade turbine of *β* = 30^o^, the power numbers *Ne* decrease slightly only with the increase in the parameter *p*/*H*, i.e. with the decrease in the baffle length *L*. In the case of the pitched blade turbine of *β* = 45^o^, power numbers *Ne* decrease about two times within the range *p*/*H* ϵ < 0.7; 1 >, and they are practically constant within the *p*/*H* ϵ < 0; 0.7). In the agitated vessel with the pitched blade turbine of *β* = 60^o^, power consumption decreases about three times within the range of the geometrical parameters *p*/*H* ϵ < 0.7; 1 >. The function *Ne* = *f*(*p*/*H*) for the turbine with the flat blades (= 90^o^) shows that power numbers strongly decrease within the ranges of the parameter *p*/*H* ϵ < 0; 0.2) and *p*/*H* ϵ < 0.7; 1 >, whereas the values of the *Ne* differ slightly only within the *p*/*H* ϵ < 0.2; 0.7 >.

**Fig. 12:**
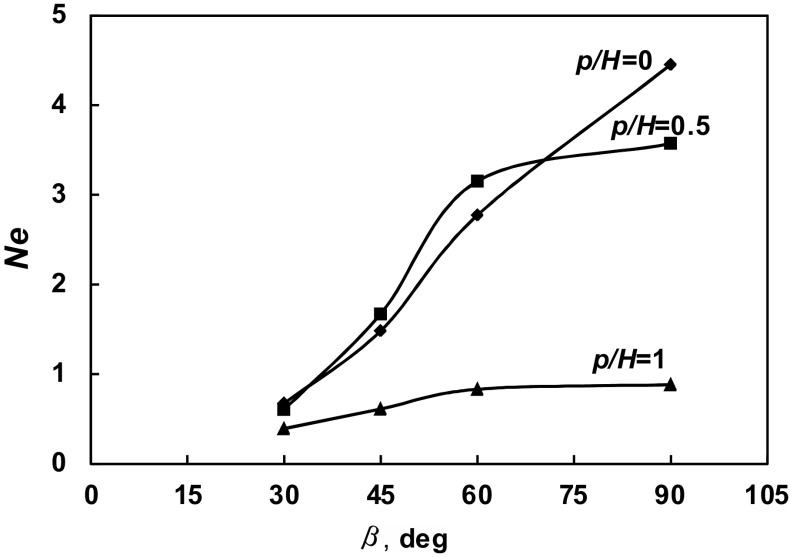
Dependence *Ne* = *f* (*β*) for different geometrical parameters *p/H* = 0; 0.5; 1

The dependence of the power number *Ne* on the angle *β* of the pitched blade turbine is presented in Fig. 12 for the given values of the geometrical parameters *p*/*H*. The strongest increase in the power number with the *β* is observed for the agitated vessel equipped with the pitched blade turbine and planar baffles of the length *L* = *H*, i.e. for the parameter *p*/*H* = 0. In the agitated vessel without baffles (*p*/*H* = 1), power number *Ne* increases about two times within the range of the angle *β* ϵ < 30^o^; 90^o^ >. For the agitated vessel with the short baffles of the length *L* = 0.5*H* (*p*/*H* = 0.5), the power numbers *Ne* are comparable to those for the vessel equipped with standard baffles (*L* = *H*) within the range of the angle *β* ϵ < 30^o^; 70^o^ >, whereas they are about 20% lower for the *β* = 90^o^.

**Fig. 13:**
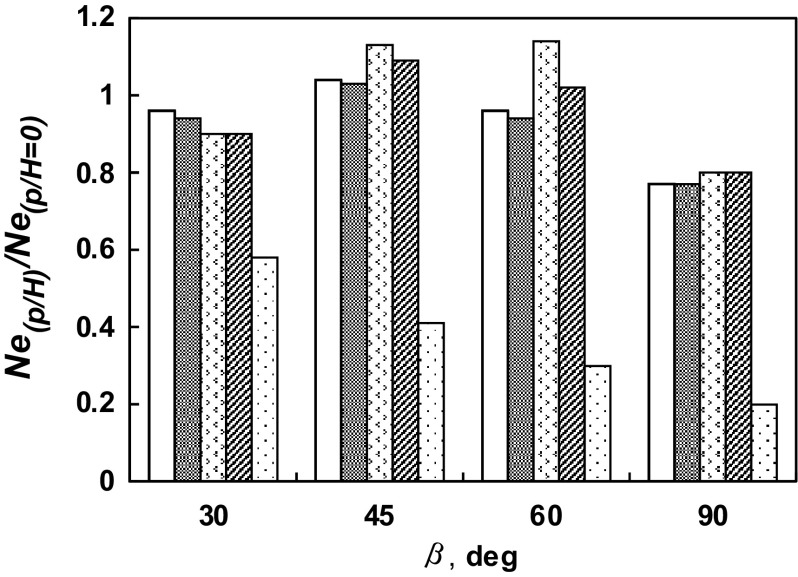
Dependence *Ne*_*(p/H)*_ */Ne*_*(p/H* = *0)*_ = *f* (*β*) for different length of the short baffles: *p/H* = 0.17( ); *p/H* = 0.33( ); *p/H* = 0.5( ); *p/H* = 0.67( ); *p/H* = 1( )

The ratio of the power number for the agitated vessel equipped with the short baffles *Ne*_(*p/H*)_ to the power number for the vessel with the standard baffles *Ne*_(*p/H* = 0)_ was also determined. The dependence of the relative power numbers as function of the angle of the blade inclination to the horizontal is shown in Fig. 13 for the different parameter *p*/*H*. The comparison of the data for the agitated vessels with standard baffles and without baffles shows that function *Ne*_(*p/H* = 1)_/*Ne*_(*p/H* = 0)_ = *f* (*β*) increases within the limits (0.2; 0.6) as the angle *β* of the turbine blade changes from the 90 to the 30 degree. An analogous analysis was carried out for the agitated vessels equipped with standard and short baffles. In this case, the functions *Ne*_(*p/H*)_/*Ne*_(*p/H* = 0)_ = *f* (*β*) are approximately equal to ≈ 0.8 or ≈ 0.9 for the limiting values of the angle *β*, i.e. 90^o^ and 30^o^, respectively. The values, which correspond to the short baffles of the length *L* = 0.5*H* (*p*/*H* = 0.5), have the maximum approximately for the *β* ≈ 45^o^ and 60^o^ and are located the highest in the graph Ne_(*p/H*)_/Ne_(*p/H* = 0)_ = *f* (*β*) in comparison with the other values within the limits of the geometrical parameter *p*/*H* ∈ < 0.17; 0.67 >.

The dependences of the power number *Ne* on the pitch *β* of the turbine blade within the range of *β* ϵ < *π*/6; *π*/2 > for different geometrical parameters *p*/*H* were described by means of the following equations: 

for the agitated vessel with standard baffles (*p*/*H* = 0; *L*/*H* = 1),

4 $$Ne_{{\left( {p/H = 0} \right)}} = \left( { - 1.3407\beta^{2} + 3.0758\beta + 1.3071} \right)\beta \sin \beta ,$$

for the agitated vessel with short baffles of length *L* = 0.5*H* (*p*/*H* = 0.5; *L*/*H* = 0.5)

5 $$Ne_{{\left( {p/H = 0.5} \right)}} = \left( { - 4.3364\beta^{2} + 9.0648\beta - 1.2641} \right)\beta \sin \beta ,$$

and for the agitated vessel without baffles (*p*/*H* = 1; *L*/*H* = 0)

6 $$Ne_{{\left( {p/H = 1} \right)}} = 0.875 \, \sin \beta ,$$

where *β* ∈ < *π*/6; *π*/2 > should be expressed by radian. Equations (4, 5, 6) approximate the results of the measurements with mean relative errors ± 6%.

The effects of the geometrical parameter *p*/*H* on the power number *Ne* for the agitated vessel equipped with the pitched bade turbine of the pitch *β* were also mathematically described for each of four impellers, i.e.: for pitched blade turbine with the *β* = 30^o^

7 $$Ne_{{\left( {\beta = 30 \, \deg } \right)}} = - 0.2858\left( {p/H} \right)^{2} + 0.0085\left( {p/H} \right) + 0.6676,$$

within the *p*/*H* ϵ < 0; 1 >.For pitched blade turbine with the *β* = 45^o^

8 $$Ne_{{\left( {\beta \, = \, 45 \, \deg } \right)}} = - 2.517\left( {p/H} \right)^{2} + 1.6509\left( {p/H} \right) + 1.4764,$$

within the *p*/*H* ϵ < 0; 1 >. For pitched blade turbine with the *β*  = 60^o^

9 $$Ne_{{\left( {\beta = \, 60 \, \deg } \right)}} = 7.754\left( {p/H} \right)^{2} - 3.1234\left( {p/H} \right) + 2.7731,$$

within the *p*/*H* ϵ < 0; 0.5 > and

10 $$Ne_{{\left( {\beta = \, 60 \, \deg } \right)}} = - 8.1232\left( {p/H} \right)^{2} + 7.5426\left( {p/H} \right) + 1.4094,$$

within the *p*/*H* ϵ (0.5; 1 >. For pitched blade turbine with the  *β* = 90^o^

11 $$Ne_{{\left( {\beta = \, 90 \, \deg } \right)}} = - 32.2774\left( {p/H} \right)^{3} + 34.5686\left( {p/H} \right)^{2} - 10.959\left( {p/H} \right) + 4.446,$$

within the *p*/*H* ϵ < 0; 0.5 > and

12 $$Ne_{{\left( {\beta = \, 90 \, \deg } \right)}} = - 15.8273\left( {p/H} \right)^{2} + 18.343\left( {p/H} \right) - 1.6407,$$

within the *p*/*H* ϵ (0.5; 1 >.

Mean relative errors of Eqs. (7, 9–12) were estimated as ± 4%, whereas Eq. (8) approximates results of the measurements with error ± 6%.

Experimentally obtained power numbers *Ne* for the agitated vessel with standard baffles, compared to those from the literature data (Karcz 1991; Bates et al. 1963) in Table 2, are seen to be in sufficient agreement.

**Table 2:** Comparison of the literature data with the experimental power numbers *Ne* obtained for the agitated vessel with standard baffles (*p/H* = 0)

No.	*β*/deg	Literature	Data		Experimental results	
		Refs.	*D*/m	*Ne*	*D*/m	*Ne*
1	90	Karcz (1991)	0.45	4.31	0.6	4.45
		Bates et al. (1963)	0.3	4		
2	60	Karcz (1991)	0.45	2.79	0.6	2.77
3	45	Karcz (1991)	0.45	1.67	0.6	1.48
		Bates et al. (1963)	0.3	1.3		
4	30	Karcz (1991)	0.45	0.75	0.6	0.67

## Conclusions

The results of the experimental studies of the power consumption for the agitated vessel equipped with the pitched blade turbine and short planar baffles have revealed that within the range of the taken measurements:

1. Power number *Ne* depends on the length *L* of the baffle and the angle *β* of the blade inclination of the turbine.
2. For the assumed value of the angle *β*, the function *Ne* = *f*(*L*/*H*) decreases with the decrease in the baffle length *L* (i.e. with the increase in the parameter *p*). The greatest differences between power numbers *Ne* were observed for the turbine with the flat blades (*β* = 90^o^).
3. For the assumed value of the baffle length *L*, the function *Ne* = f(*β*) increases with the increase in the angle *β* of the inclination of the impeller blade. The greatest effect was found for the agitated vessel equipped with the standard baffles (*L* = *H*), whereas the power numbers are dependent on the *β* for the agitated vessel without baffles the least of all.

## Symbols

*B*: Width of the baffle (m)
*b*: Width of the agitator blade (m)
*D*: Inner diameter of the agitated vessel (m)
*d*: Diameter of the agitator (m)
*H*: Liquid height in the vessel (m)
*h*: Distance between agitator and bottom of the vessel (m)
*J*: Number of baffles
*L*: Length of the baffle (m)
*M*: Torque (Nm)
*Ne*: Power number (= *P*/*n* ^3^ *d* ^5^ *ρ*)
*n*: Agitator speed (s^−1^)
*n*_JD_: Just draw down agitator speed (s^−1^)
*P*: Power consumption (W)
*p*: Distance between lower edge of the baffle and bottom of the vessel (m)
Re: Reynolds number for mixing (= nd^2^ *ρ*/*η*)
*Z*: Number of turbine blades

## Greek letters

*β*: Angle of the inclination of the turbine blade to the horizontal (deg, rad)
*η*: Dynamic viscosity of the liquid (kg m^−1^ s^−1^)
*ρ*: Liquid density (kg m^−3^)
